# A Pyrazolo[3,4-*d*]pyrimidine Compound Reduces Cell Viability and Induces Apoptosis in Different Hematological Malignancies

**DOI:** 10.3389/fphar.2016.00416

**Published:** 2016-11-07

**Authors:** Ilaria Laurenzana, Antonella Caivano, Francesco La Rocca, Stefania Trino, Luciana De Luca, Francesca D’Alessio, Silvia Schenone, Geppino Falco, Maurizio Botta, Luigi Del Vecchio, Pellegrino Musto

**Affiliations:** ^1^Laboratory of Preclinical and Translational Research, IRCCS - Referral Cancer Center of Basilicata (CROB), Rionero in VulturePotenza, Italy; ^2^Biotecnologie Avanzate s.c.a.r.l., CEINGENapoli, Italy; ^3^Department of Pharmacy, University of GenoaGenoa, Italy; ^4^Department of Biology, University of Naples Federico IINaples, Italy; ^5^Department of Biotechnology, Chemistry and Pharmacy, University of SienaSiena, Italy; ^6^Department of Molecular Medicine and Medical Biotechnologies, University of Naples Federico IINaples, Italy; ^7^Scientific Direction, IRCCS – Referral Cancer Center of Basilicata (CROB), Rionero in VulturePotenza, Italy

**Keywords:** targeted therapies, Src kinase inhibitor, pyrazolo[3, 4-*d*]pyrimidine compound, Fyn tyrosine kinase, hematological malignancies

## Abstract

Molecular targeted therapies are based upon drugs acting on tumors by interfering with specific targets involved in growth and spread of cancer. Many targeted therapies were approved by Food and Drug Administration as standard treatment, others were introduced into preclinical or clinical studies on hematological malignancies (HMs). The development of drug-resistance in some HMs and the lack of effective treatments in other ones emphasized the need for searching new molecular targets and therapeutic agents. The aim of this study was to evaluate the effects of 4c pyrazolo[3,4-*d*]pyrimidine compound, a Src inhibitor, on lymphoid and myeloid neoplasms. Here, we demonstrated its ability to reduce cell viability, induce apoptosis and cell cycle arrest in lymphoid cell lines such as Jurkat, SKMM1, Derl-2/7, and myeloid cell lines, such as Jurl-MK1. Moreover, we reported a high expression of a Src kinase, Fyn, in these cell lines compared to healthy subjects. This study was a starting point to investigate 4c pyrazolo[3,4-*d*]pyrimidine compound as a drug for HMs and Src kinases as its potential molecular targets.

## Introdution

Targeted cancer therapies are “drugs” designed to interfere with specific molecules necessary for tumor growth and progression. These agents are broadly classified in monoclonal antibodies and small molecules. The first are generally directed against antigens expressed on neoplastic cell surface, the second are usually designed to interfere with the enzymatic activity of the target protein. A primary goal of these kind of therapies is to fight cancer cells with more precision while to do less damage to normal cells.

The approval of imatinib mesylate for the treatment of chronic myeloid leukemia (CML) and its high success rate supported the extensive efforts to develop novel molecularly targeted therapies for hematologic malignancies (Cierpicki and Grembecka, 2015). On the other hand, the development of drug-resistance in some hematological tumors, for example in CML (An et al., 2010), and the lack of effective treatments in other ones (Martelli et al., 2012; Yang and Lin, 2015; Zinzani et al., 2015), addressed researchers to the study of new molecular targets and innovative therapeutic agents.

A lot of new compounds have been synthesized, investigated, and introduced into pre-clinical and clinical studies in hematological malignancies (HMs; Korycka-Wołowiec et al., 2015). The majority of small molecule block activity of protein kinases, including FMS-like tyrosine kinase 3 (FLT3; Levis, 2013; Plawny and Rie, 2014), Aurora kinase (Garcia-Manero et al., 2015), JAK1/2 (Pinilla-Ibarz et al., 2016), Akt, mTOR (Cierpicki and Grembecka, 2015) and Src kinases (Roskoski, 2015b).

The Src family protein tyrosine kinases (SFKs) are non-receptor intracellular kinases known to have diverse and important regulatory roles in both normal hematopoiesis and leukemogenesis. There are eight members of this family in mammals and they are structurally related, except for the unique domain: Src, Lyn, Hck, Fyn, Yes, Blk, Fgr, and Lck (Ku et al., 2015). SFKs represent appealing targets for cancer therapy because of their aberrant activation in many human cancer types and their key role in controlling several processes, such as proliferation, apoptosis, migration, and angiogenesis, whose deregulation underlies cancer development and progression (Yeatman, 2004).

Recently we synthesized new pyrazolo[3,4-*d*]pyrimidine library of SFKs inhibitors, which entered in ATP binding site and showed antiproliferative and pro-apoptotic action in several tumor types such as CML and in Burkitt lymphoma (Schenone et al., 2010; Cozzi et al., 2012; Radi et al., 2013; Tintori et al., 2013). Moreover, we demonstrated that 4c pyrazolo[3,4-*d*]pyrimidine compound was able to reduce cell viability, induce apoptosis and cell cycle arrest in a rare natural killer (NK) cell leukemia and in CML. In particular, it reduced phosphorylation of Fyn kinase in these neoplasms (Tintori et al., 2015; Laurenzana et al., 2016).

Fyn kinase is able to interact with several proteins and participates in different cellular pathways, both in physiological and pathological situations. It has been demonstrated that it is involved in the regulation of T-cell development and activation, factor and cytokine receptor signaling, cell–cell adhesion, integrin-mediated signaling, ion channel function, platelet activation, T- and B-cell receptor signaling, axon guidance, mitosis, differentiation of NK cells (Palacios and Weiss, 2004; Salmond et al., 2009; Saito et al., 2010).

The aim of this study was to analyze the effects of 4c compound on viability of cell lines derived from different lymphoid and myeloid neoplasms and to report Fyn expression analysis in these tumors compared to healthy subjects.

## Materials and Methods

### Cell Lines and Chemical

Peripheral blood samples were obtained from healthy donors (HDs). The study was approved by the Ethics Committee of IRCCS-CROB (Prot. 3725; 07/02/2008) and all subjects gave informed consent according to the Declaration of Helsinki. Peripheral blood mononuclear cells from HDs (HD-PBMCs) were isolated by Ficoll-hypaque gradient separation.

Both human lymphoid and myeloid cell lines were purchased from Leibniz-Institut DSMZ – Deutsche Sammlung von Mikroorganismen und Zellkulturen GmbH or from American Type Culture Collection and cultured as follows. Jurkat (acute T cell leukemia), Derl-2 and Derl-7 (T cell lymphoma), NALM-6 and BV173 (acute lymphoblastic leukemia), Jurl-MK1 (CML), NB4 and HL-60 (acute promyelocytic leukemia), Kasumi-1 (acute myeloid leukemia) cell lines were cultured in RPMI 1640 (Gibco, Life technologies, Carlsbad, CA, USA) supplemented with 20% fetal bovine serum (FBS, Gibco) and 1% of penicillin-streptomycin (pen/strep, Gibco). For Derl-2 and Derl-7, 20 ng/ml interleukin-2 (IL-2, Miltenyi Biotec, Auburn, CA, USA) was added at the previous indicated medium. OCI-AML3 cell line (acute myeloid leukemia) was cultured in Dulbecco’s Modified Eagle Medium (DMEM, Gibco) supplemented with 20% FBS, 1% of pen/strep. SKMM1 cells (multiple myeloma) were cultured in Iscove’s Modified Dulbecco’s Medium (IMDM, Gibco) supplemented with 10% FBS, 1% of pen/strep. All cell lines were maintained in incubator at 37°C and 5% CO_2_.

4c pyrazolo[3,4-*d*]pyrimidine compound, given by Lead Discovery Siena s.r.l. (patent: WO2016066755), was dissolved in dimethyl sulfoxide (DMSO, Sigma-Aldrich, St Louis, MO, USA) and diluted in FBS for cell treatments.

### Western Blotting (WB) Analysis

Cells were lysed as previously reported (Trino et al., 2016). Sixty micrograms was subjected to sodium dodecyl sulfate polyacrylamide gel electrophoresis on a 10% gel under reducing conditions and then electrotransferred onto a polyvinylidene difluoride membranes using Trans Blot Turbo Transfer System (BioRad, Hercules, CA, USA). Membranes were probed with primary antibodies directed against Fyn and β-actin (Cell Signaling, Beverly, MA, USA), then incubated with secondary antibody (horseradish peroxidase-conjugated goat anti-mouse or anti-rabbit; Cell Signaling). Immune complexes were detected by ECL chemiluminescence system (Bio-Rad Laboratories), as recommended by the manufacturer. Densitometric analysis was performed using Bio-Rad Image Lab 4.1 software. The intensity of bands of all proteins was normalized to the β-actin signal.

### Cell Viability

All cell lines were seeded into 96-well plates (3 × 10^4^ cells/100 μl) and incubated with 4c compound at increasing concentrations (1, 5, 10, 15 μM). HD-PBMCs were incubated with 15 μM of 4c compound. Treatment was carried out for 24, 48, and 72 h. Cells treated with DMSO vehicle were used as control. Cell viability was determined using the CellTiter 96 Aqueous One Solution assay kit (MTS, Promega, Madison, WI, USA). The optical density was measured at 492 nm. Cellular viability was calculated as percentage of viable cells compared with control. All experiments were conducted in triplicate. EC_50_ values were obtained by GraphPad Prism (GraphPad Prism, San Diego, CA, USA).

### Functional Tests

Cells were seeded at 3 × 10^5^ cells/ml cell density. Jurkat were treated with 2 μM of 4c compound for 24, 48, and 72 h; Derl-2 and Derl-7 with 4 μM of 4c compound for 72 h; while Jurl-MK1 with 1 μM of 4c compound for 72 h. All cell lines were incubated with DMSO vehicle as control. After 4c compound treatment cells were used in:

#### Apoptosis Assay

Apoptosis was evaluated by cytometric analysis of Annexin V and Propidium Iodide (PI)-stained cells using fluorescein isothiocyanate (FITC) Annexin V Apoptosis Detection kit I (BD) as previously reported (De Luca et al., 2016). Cells were acquired using FACSCalibur flow cytometer and analyzed by CellQuest Pro software (BD). Single positive for Annexin V and double positive for Annexin V and PI cells were interpreted as signs of early and late phases of apoptosis, respectively.

#### Cell Cycle Analysis

After treatment cells were fixed in cold ethanol 70% for 1 h, then labeled with PI/RNase staining solution for 30 min. Samples were acquired by FACSCalibur (BD). Data were analyzed by ModFit LT Software (Verity Software House).

### Statistical Analysis

Statistical significance was determined using a paired *t*-test by GraphPad Prism. All error bars represent standard deviation (SD) of the mean. A *P*-value ≤ 0.05 was accepted as statistically significant.

## Results

### 4c Pyrazolo[3,4-*d*]pyrimidine Compound Reduced Cell Viability in Hematological Malignancies

We treated 3 HD-PBMCs, 6 lymphoid and 5 myeloid cell lines (**Table 1**) with 4c pyrazolo[3-4,*d*]pyrimidine compound or with DMSO vehicle control at increasing concentrations (1–15 μM) from 24 to 72 h to verify its effect on cell viability.

**Table 1:** Hematological malignancies (HM) cell lines analyzed.

	Cell lines	Disease
Lymphoid Neoplasms	Jurkat	Acute T cell leukemia
	SKMM1	Multiple myeloma
	Derl-2	T cell lymphoma
	Derl-7	T cell lymphoma
	BV-173	Acute lymphoblastic leukemia
	NALM-6	Acute lymphoblastic leukemia
Myeloid Neoplasms	Jurl-MK1	Chronic myeloid leukemia
	NB-4	Acute promyelocytic leukemia
	HL-60	Acute promyelocytic leukemia
	OCI-AML3	Acute myeloid leukemia
	Kasumi-1	Acute myeloid leukemia

**FIGURE 1:**
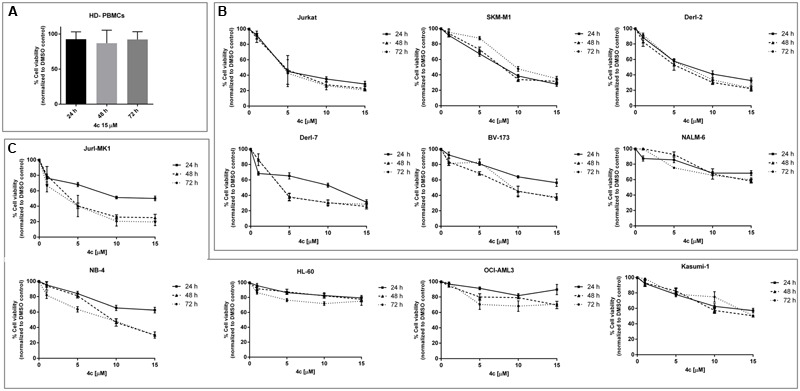
**Cell viability assay after 4c compound treatment in hematological malignancies.** Viability of three healthy donors-peripheral blood mononuclear cells (HD-PBMCs) **(A)**, six lymphoid **(B)**, and five myeloid **(C)** cell lines was evaluated by MTS assay after treatment with 4c compound at different concentration (1, 5, 10, and 15 μM) for 24, 48, and 72 h. Results are expressed as percent of cell viability normalized to DMSO-treated control cells. The bar-graphs represent mean with SD from three independent experiments.

First of all, we observed that 4c compound had negligible effect on HD-PBMCs at 15 μM in a time course, from 24 to 72 h (**Figure 1A**). In the context of lymphoid malignancies (**Figure 1B**), Jurkat, SKMM1 and Derl-2 cell lines showed a wide reduction of cell viability (about -50%) already at 5 μM and it increased at higher concentrations of 4c compound, reaching a reduction of cell viability of 60–70% at 10 μM. This effect was observed at 24 h and remained constant at other time points. Derl-7 and BV-173 showed a dose-dependent viability reduction after treatment with 4c compound with the higher effect at 48 and 72 h, -25 and -60% at 15 μM, respectively. On NALM-6 cell line, we observed a reduction of viability of 40% at 15 μM of 4c compound at all time points.

In the context of myeloid malignancies (**Figure 1C**), Jurl-MK1 and NB-4 showed a dose-dependent reduction of viability after 4c compound treatment, especially at 48 and 72 h.

HL-60 and OCI-AML3 showed 20 and 30% reduction of cell viability, respectively, after 15 μM 4c treatment at all time points. Kasumi-1 showed a decrease of viability at increasing concentration of 4c compound at all time points; the reduction of 50% was observed at 15 μM at 48 and 72 h.

We calculated 4c compound EC_50_ values for all cell lines at 24, 48, and 72 h (**Table 2**). For lymphoid neoplasms, Jurkat cells showed EC_50_ around 4 μM at 24, 48, and 72 h; SKMM1showed EC_50_ = 7.7 μM at 24 h which increased at 48 and 72 h; Derl-2, Derl-7 and BV-173 showed a decrease of EC_50_ values over time. For NALM-6 EC_50_ value was much greater than the highest concentration used. For myeloid malignancies, Jurl-MK1 showed EC_50_ > 15 μM at 24 h but it considerably decreased at 2.5 μM at 48 and 72 h. The same trend was observed in NB-4 cells, in which EC_50_ was >15 μM at 24 h and it decreased at 48 and 72 h at 7.2 and 5.9 μM, respectively. HL-60 and OCI-AML3 had EC_50_ higher than 15 μM at all time points. Kasumi-1 showed EC_50_ = 15 μM at 48 and 72 h.

**Table 2:** EC_50_ values calculated in Healthy donors-peripheral blood mononuclear cells (HD-PBMCs) and in both lymphoid and myeloid cell lines after 4c compound treatment.

	Cells	EC_50_ [μM]		
		24 h	48 h	72 h
	HD-PBMCs	>15	>15	>15
Lymphoid Neoplasms	Jurkat	4.6	4	3.8
	SKMM1	7.7	8.11	10.8
	Derl-2	7.5	5.9	6.2
	Derl-7	12.3	4.7	4.7
	BV-173	>15	6.6	7.15
	NALM-6	>15	>15	>15
Myeloid Neoplasms	Jurl-MK1	>15	2.5	2.4
	NB-4	>15	7.2	5.9
	HL-60	>15	>15	>15
	OCI-AML3	>15	>15	>15
	Kasumi-1	>15	15	15

### 4c Pyrazolo[3,4-*d*]pyrimidine Compound Increased Apoptosis in Jurkat, Derl-2, Derl-7, and Jurl-MK1 Cells

**FIGURE 2:**
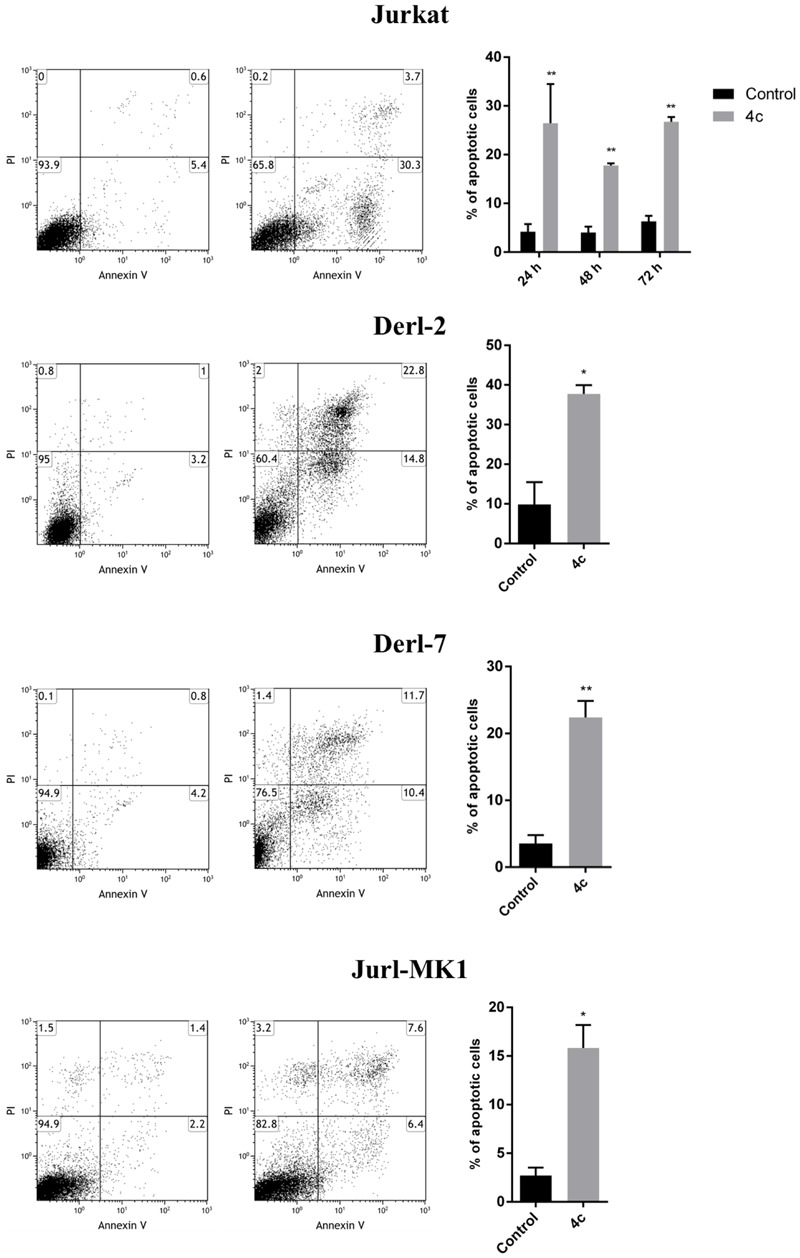
**Apoptosis analysis in hematological malignancies after 4c compound treatment.** Apoptosis was evaluated by flow cytometer in Jurkat cell line after 4c compound or DMSO vehicle treatment at 2 μM for 24, 48, and 72 h; in Derl-2 and Derl-7 after treatment at 4 μM for 72 h; in Jurl-MK1 after treatment at 1 μM for 72 h. Dot plots show a single representative experiment, the bar-graphs represent mean with SD from three independent experiments. ^∗^ and ^∗∗^ indicated a *P*-value minor than 0.05 and 0.01, respectively.

To further investigate cell death mechanism induced after treatment, we performed apoptosis on Jurkat, Derl-2, Derl-7, and Jurl-MK1 cell lines by cytometric analysis of Annexin V/PI (**Figure 2**). Jurkat cells were treated with 2 μM of 4c compound for 24, 48, and 72 h. After treatment we observed a significant increase of apoptotic cells at all time points respect to their control, 34% at 24 h, 18% at 48 h, and 27% at 72 h (*p* < 0.01). The treatment with 4 μM of 4c compound for 72 h on Derl-2 and Derl-7 induced an increased apoptosis respect to their control (40% *p* < 0.05 and 23% *p* < 0.01, respectively). Jurl-MK1 cells, after incubation with 1 μM of 4c compound for 72 h, showed an increased apoptosis rate respect to control (18%; *p* < 0.05).

### 4c Pyrazolo[3,4-*d*]pyrimidine Compound Induced Cell Cycle Arrest in Jurkat, Derl-2, Derl-7, and Jurl-MK1 Cells

**FIGURE 3:**
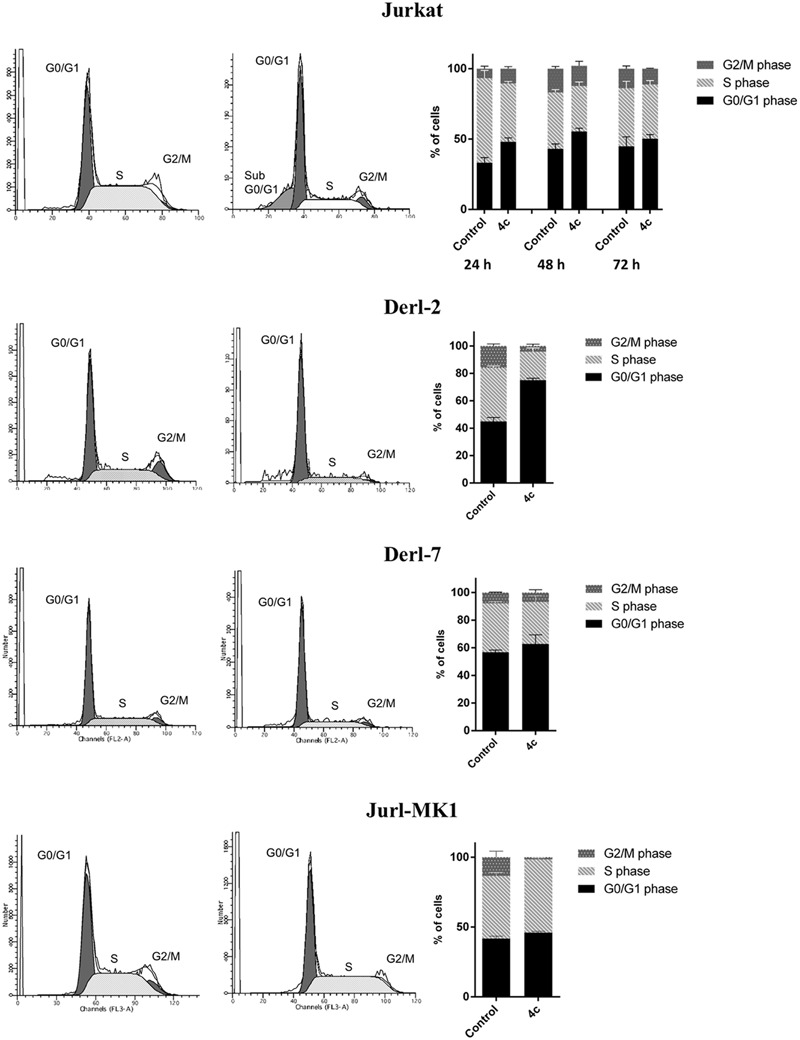
**Cell cycle analysis in hematological malignancies after 4c compound treatment.** Cell cycle analysis was evaluated by flow cytometer in Jurkat cell line after 4c compound or DMSO vehicle treatment at 2 μM for 24, 48, and 72 h; in Derl-2 and Derl-7 after treatment at 4 μM for 72 h; in Jurl-MK1 after treatment at 1 μM for 72 h. Histogram plots show a single representative experiment, the bar-graphs represent mean with SD from three independent experiments.

We also performed cell cycle analysis on Jurkat, Derl-2, Derl-7, and Jurl-MK1 cell lines by cytometric analysis of PI (**Figure 3**). Jurkat were treated with 2 μM of 4c compound for 24, 48, and 72 h; Derl-2 and Derl-7 with 4 μM of 4c at for 72 h; while Jurl-MK1 with 1 μM of 4c compound for 72 h. Jurkat showed cell cycle arrest in G0/G1 phase in all time points, especially at 24 h, respect to control. Derl-2 displayed a strong arrest in G0/G1 phase, whereas Derl-7 manifested a negligible effect on cell cycle after treatment, respect to control. Jurl-MK1 showed a small increase of G0/G1 phase and a larger raise of percent of cells in S phase.

### Fyn Is Over-Expressed in Hematological Malignancies

**FIGURE 4:**
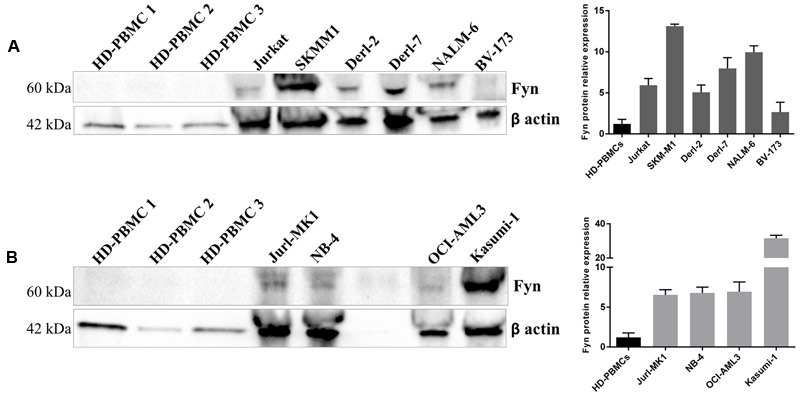
**Fyn expression in different hematological malignant cells.** Western Blotting (WB) analysis of Fyn levels in 3 HD-PBMCs compared to lymphoid cell lines **(A)** and in three HD-PBMCs compared to myeloid cell lines **(B)**. Quantification of Fyn protein levels was normalized with β-actin bands. The bar-graphs represent mean with SD from three independent experiments.

We analyzed Fyn protein expression in 3 HD-PBMCs, in lymphoid and myeloid cell lines by WB analysis. Fyn is over-expressed in lymphoid cell lines respect to HD-PBMCs; in particular, SKMM1 showed the major amount of protein, following by NALM-6, Derl-7, Jurkat, Derl-2, and BV-173. Also all myeloid Jurl-MK1, NB-4, OCI-AML3, and Kasumi-1 cells presented higher Fyn protein expression (**Figure 4**).

## Discussion

“Molecular targeted therapies” are used in the treatment of many cancers as first-line therapy (Neul et al., 2016). The first tyrosine kinase inhibitor (TKI) approved for the treatment of CML was imatinib mesylate (Cierpicki and Grembecka, 2015). Since then, over 3000 novel agents inhibiting diverse protein kinases are currently being explored preclinically and, at present, more than 130 novel TKIs are being evaluated in oncological clinical trials (Neul et al., 2016).

Recent studies on tumor pathobiology in hematological and solid cancers have revealed that heterogeneity is the major cause of poor drug efficacy and response duration (Visvader, 2011; McGranahan and Swanton, 2015; Belizário et al., 2016). Moreover, cancer cells that respond to particularly therapeutic treatment can rapidly adapt and develop drug-resistance changing their signaling pathways (Luo et al., 2009; Carragher et al., 2012; McGranahan and Swanton, 2015; Belizário et al., 2016). At this point comes the need to screen new molecular targets and innovative therapeutic agents.

Protein kinases, including SFKs, are the most attractive target structures. They are key players in signal transduction networks mediating fundamental cellular processes such as cell differentiation, proliferation, apoptosis, transcription, metabolism, and intercellular communication. During the past 15 years, was emerged that many cancers originated from dysregulation of their signaling pathways (Manning et al., 2002; Levitzki, 2013; Rask-Andersen et al., 2014; Roskoski, 2015a) and drug development has shifted toward small molecules that specifically block them.

In this context, our group developed a library of pyrazolo[3,4-*d*]pyrimidine compounds which have the ability to inhibit some of Src kinases by entering in the ATP binding site. In particular, we have previously demonstrated that 4c pyrazolo[3,4-*d*]pyrimidine compound, is able to inhibit Fyn kinase and to induce apoptosis in cells derived from NK leukemia and from CML (Tintori et al., 2015; Laurenzana et al., 2016).

In this study, we demonstrated, for the first time, the cytotoxic effect of 4c compound on HMs compared with HD-PBMCs.

First, we investigated the response of cell viability to 4c compound treatment. We have recently demonstrated that 4c compound, up to 10 μM, had no effect on HD-PBMCs cell viability at 24, 48, and 72 h (Laurenzana et al., 2016). In this study, we confirmed these data and observed that also 15 μM of 4c compound had no effect on viability of HD-PBMCs at all time points.

Interestingly, Jurkat, SKMM1, Derl-2, Derl-7, and BV-173 showed a dose-dependent reduction of cell viability and their EC_50_ values were in a range of 3–10 μM at all time points. Derl-2 and Derl-7 were established from the same patient with hepatosplenic γδ T cell lymphoma, but they showed a different phenotype: Derl-2 was TcRγδ^+^, while Derl-7 was TcRγδ^-^ (Di Noto et al., 2001). Derl-2, closer to T cells, showed a lower EC_50_ at 24 h. Within lymphoid cell lines, NALM-6 showed a lower response to 4c treatment and a reduction of 50% of cell viability at higher concentrations respect to other cells. BV-173 and NALM-6 derived both from acute lymphoblastic leukemia but showed different response to 4c compound, probably due to the clinical and biological heterogeneity of this neoplasm. BV-173, for example, expressed Philadelphia (Ph) chromosome, lacking instead in NALM-6. In the context of myeloid cell lines, we observed an EC_50_ achievement at concentration ≤15 μM at 48 and 72 h for Jurl-MK1, NB-4, and Kasumi-1 cell lines. Jurl-MK1 response could be comparable to lymphoid BV-173 cells probably due to the presence of Ph chromosome in both cell lines.

In general, lymphoid cells seemed to respond better than myeloid ones. In agreement with our data, a lot of recent studies showed that the use of TKIs, especially against Btk, Syk, and Lyn, is a promising new strategy for targeted treatment of B-cell lymphoid malignancies (Robak and Robak, 2012). Furthermore, these data on Jurl-MK1 confirmed our previously results obtained in K562 CML cell line (Tintori et al., 2015).

We decided to study in deep the viability reduction in Jurkat, Derl-2, Derl-7, and Jurl-MK1 cell lines and we demonstrated that 4c compound induced apoptosis and cell cycle arrest.

Recent studies showed that other small molecules induced cell viability reduction and apoptosis in cell lines used in our study. For example, Bucur et al. (2015) synthesized a small molecule that binds caspase 8 and enhances its activation when combined with TRAIL inducing apoptosis in Jurkat cells. Furthermore, Lin et al. (2010) demonstrated that SMI-4a, a small molecule inhibitor of Pim kinases, kill a wide range of both myeloid and lymphoid cell lines with precursor T-cell lymphoblastic leukemia/lymphoma being highly sensitive.

Since we previously observed, by *in vitro* enzymatic assay, that 4c compound inhibited different Src kinases, in particular Fyn with IC_50_ = 0.07 μM (Tintori et al., 2015), we verified the presence of Fyn protein in our HM cell lines. Interestingly, we observed that its expression is higher and variable in all cell lines compared to HD-PBMCs.

Fyn, a member of SFKs, has diverse molecular functions, including regulation of cell growth, survival, adhesion, cytoskeletal remodeling, motility, axon guidance, synaptic function, platelet activation, and T cell receptor signaling. It is involved in various aspects of the pathogenesis of different types of cancers as well as drug resistance (Elias and Ditzel, 2015) so it could be a potential therapeutic target.

In a rare NK leukemia, we demonstrated that antiproliferative activity of 4c compound was due to Fyn kinase inhibition (Laurenzana et al., 2016). Recently, Palomero et al. (2014) demonstrated that dasatinib, a multikinase inhibitor which blocks ABL1 and SRC kinases, induced dose dependent inhibition of FYN phosphorylation.

Our preliminary data indicated Fyn as a potential target of 4c compound but cannot be excluded that 4c compound might also act on other Src kinases activated in neoplastic cells. Other experiments are needed to evaluate this hypothesis.

In summary, in this study we demonstrated that 4c pyrazolo[3,4-*d*]pyrimidine compound had cytotoxic effect by inducing a reduction of cell viability and a increased apoptosis in different cell lines derived from HMs. Finally, we observed that 4c compound potential target, Fyn kinase, is over-expressed in all cell lines used. But others experiments are necessary in this fields, because this study represents a starting point to better investigate the effect of 4c pyrazolo[3,4-*d*]pyrimidine compound and the role of Src kinases in HMs.

## Author Contributions

All authors participated to the conception of the study; IL, designed the work, planned and performed experiments, performed flow cytometry analysis, analyzed data, drafted paper; AC, designed the work, planned experiments, analyzed data, drafted paper; FL, ST, LD, FD, performed experiments and analyzed data; SS and MB, designed and synthesized chemical compound; GF improved writing paper; LD and PM, designed the work, improved writing paper; all authors approved the final version of the manuscript.

## Conflict of Interest Statement

The authors declare that the research was conducted in the absence of any commercial or financial relationships that could be construed as a potential conflict of interest.
